# Feasibility of unconstrained three-material decomposition: imaging an excised human heart using a prototype silicon photon-counting CT detector

**DOI:** 10.1007/s00330-020-07017-y

**Published:** 2020-06-25

**Authors:** Fredrik Grönberg, Johan Lundberg, Martin Sjölin, Mats Persson, Robert Bujila, Hans Bornefalk, Håkan Almqvist, Staffan Holmin, Mats Danielsson

**Affiliations:** 1 Department of Physics, AlbaNova University Center, KTH Royal Institute of Technology, SE-106 91 Stockholm, Sweden; 2 Department of Clinical Neuroscience, Karolinska Institutet and the Department of Neuroradiology, Karolinska University Hospital, Stockholm, Sweden; 3 Department of Bioengineering, Stanford University, Stanford, CA 94305 USA; 4 Medical Radiation Physics and Nuclear Medicine, Karolinska University Hospital, Stockholm, Sweden

**Keywords:** Tomography, X-ray computed, Contrast media, Plaque, atherosclerotic, Algorithms, Phantoms, imaging

## Abstract

**Rationale and objectives:**

The purpose of this study was to evaluate the feasibility of unconstrained three-material decomposition in a human tissue specimen containing iodinated contrast agent, using an experimental multi-bin photon-counting silicon detector. It was further to evaluate potential added clinical value compared to a 1st-generation state-of-the-art dual-energy computed tomography system.

**Materials and methods:**

A prototype photon-counting silicon detector in a bench-top setup for x-ray tomographic imaging was calibrated using a multi-material calibration phantom. A heart with calcified plaque was obtained from a deceased patient, and the coronary arteries were injected with an iodinated contrast agent mixed with gelatin. The heart was imaged in the experimental setup and on a 1st-generation state-of-the-art dual-energy computed tomography system. Projection-based three-material decomposition without any constraints was performed with the photon-counting detector data, and the resulting images were compared with those obtained from the dual-energy system.

**Results:**

The photon-counting detector images show better separation of iodine and calcium compared to the dual-energy images. Additional experiments confirmed that unbiased estimates of soft tissue, calcium, and iodine could be achieved without any constraints.

**Conclusion:**

The proposed experimental system could provide added clinical value compared to current dual-energy systems for imaging tasks where mix-up of iodine and calcium is an issue, and the anatomy is sufficiently small to allow iodine to be differentiated from calcium. Considering its previously shown count rate capability, these results show promise for future integration of this detector in a clinical CT scanner.

**Key Points:**

*• Spectral photon-counting detectors can solve some of the fundamental problems with conventional single-energy CT.*

*• Dual-energy methods can be used to differentiate iodine and calcium, but to do so must rely on constraints, since solving for three unknowns with only two measurements is not possible. Photon-counting detectors can improve upon these methods by allowing unconstrained three-material decomposition.*

*• A prototype photon-counting silicon detector with high count rate capability allows performing unconstrained three-material decomposition and qualitatively shows better differentiation of iodine and calcium than dual-energy CT.*

## Introduction

It is well known that the development efforts of photon-counting detectors (PCDs) for computed tomography (CT) are motivated by a desire to resolve some of the fundamental problems with single-energy CT using energy-integrating detectors. These include limited spatial resolution, limited low-dose performance [1], and non-quantitative imaging [2]. The current proposition of addressing the issue of non-quantitative imaging clinically is dual-energy CT (DECT). The use of DECT can provide quantitative tissue-specific information as a decomposition into two basis materials [3].

It is also well known that the linear attenuation coefficient of human tissue is well characterized by two basis materials and that a third basis function can be used to capture the linear attenuation of heavier elements with k-edges in the spectrum. Since dual-energy systems only provide measurements at two effective energies, some kind of constraint (typically mass preservation) needs to be imposed in order to estimate a third component. Although this enables visualization of k-edge materials with dual-energy systems, any deviation in the imaged object from the assumptions of the constraint results in bias in the decomposition [4], which in practice can result in poor differentiation of iodine and calcium.

Iodine, used in the majority of CT scans, has the k-edge at 33 keV which is generally considered too low to allow k-edge imaging due to the low fraction of x-rays below this energy after attenuation through a patient [2, 5, 6]. Instead, contrast agents such as gadolinium and gold have been proposed. Iodine content can be determined with PCDs by using constraints, but like for dual-energy methods, this can lead to bias.

The demand for and general trend towards non-invasive coronary plaque diagnostics are evident in the introduction of, e.g., coronary artery calcium score CT as opposed to angiography. There is a large patient population that presents with acute chest pain, normal initial biochemical markers, and normal or non-diagnostic electrocardiogram [7, 8] that could potentially benefit from improved coronary plaque diagnostics since absence of stenosis and/or primarily calcified plaque has been shown to be a good predictor of low-risk individuals [9, 10]. However, the atherosclerotic disease is a heterogeneous process with different stages and there is an inter-individual difference in risk and possibly even intra-individual risk [11]. Therefore, increasing imaging efforts are made to find patients with high-risk/vulnerable plaques [12, 13]. One such approach is to try and better differentiate calcium and iodine using spectral photon-counting CT.

There are a number of PCD systems currently being evaluated on phantoms and human volunteers, based on either cadmium telluride [6, 14–18] or silicon [19–21], and spectral photon-counting CT is expected to be clinically available within the next 5–10 years. One of the challenges driving the design of these detectors is how to handle pulse pileup [22] at high flux. Silicon detectors have a technical advantage that allows for handling high count rates without degrading the energy resolution [23, 24]. It has been shown in simulation that silicon detectors can, depending on the system design, perform better than cadmium-based detectors on spectral imaging tasks such as iodine quantification [25].

We want to challenge the view that iodine cannot be more accurately decomposed in the presence of calcium with PCDs than with dual-energy methods ([6], p. 32), by proposing three-material decomposition as an application for imaging tasks where better separation of calcium and iodine is feasible and would add clinical value. We have in previous publications shown that decomposition into more than two basis materials is feasible in phantom studies with this detector [20, 21]. This has also been demonstrated with other PCDs with phantoms [26–31], anatomical specimens [27, 28, 32], and animal models [33, 34]. The purpose of this work is to provide proof of concept that unbiased three-material decomposition with iodine can be achieved in a clinical specimen with human tissue with an experimental photon-counting silicon detector in a bench-top setup. To this end, we image an ex vivo excised heart with calcified plaque to evaluate the potential for added clinical value for imaging tasks where differentiation of iodine and calcium is of clinical relevance.

## Materials and methods

### Experimental setup

**Fig. 1:**
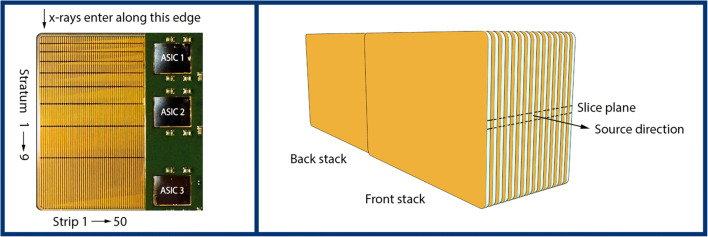
Left: experimental PCD sensor with edge-on multi-strata design. Right: illustration of assembled detector module. Sensors are assembled in two stacks to obtain full geometric efficiency

The experimental PCD is a silicon-strip detector with an edge-on multi-strata design (Fig. 1) [19–21]. The multiple strata enable the detector to operate at clinical CT count rates since the total count rate is divided among the dedicated electronics in each stratum [23]. Compared to cadmium-based detectors, this allows for handling of high count rates without reducing the pixel size to the degree that charge sharing becomes a dominating effect that reduces the spectral performance of the detector. Each pixel of the PCD consists of nine strata, each connected to a channel of a photon-counting application-specific integrated circuit (ASIC), which sorts photon counts into eight energy bins with configurable thresholds [35]. The detector module has 88 × 50 pixels that are 0.5 mm (in slice) × 0.4 mm (out of slice) at the detector.

The detector module was mounted in a bench-top setup with a fixed anode x-ray tube (COMET XRS-160, COMET Group), at a source-to-detector distance of approximately 1000 mm. A tube voltage of 120 kV, a focal spot size of 0.4 mm, and a tube current of 6 mA was used for all the experiments. The x-ray beam was filtered with 2.13 mm of aluminum and 3.5 mm of polymethyl methacrylate and was collimated to approximately 44 mm (in slice) × 20 mm (out of slice) at the detector, i.e., slightly larger than the detector module.

Objects were imaged on a rotation stage mounted at a source-to-isocenter distance of 540 mm, yielding a pixel size of 0.270 mm (in slice) × 0.216 mm (out of slice) at the isocenter. Since the detector was smaller than the imaged objects, a full field of view was achieved by translating the rotation stage.

### Calibration

The detector pixels were calibrated using a multi-material calibration phantom and a procedure suggested in [36]. The calibration phantom consisted of two plastic step wedges and three contrast agent containers. The step wedges were made of polyethylene (PE) and polyvinylchloride (PVC). These materials span the set of linear attenuation coefficients of human tissue with positive coefficients [37]. They are useful for calibration since they allow for transmission measurements mimicking any combination of tissues found in the human body. The step wedges contained steps of 0, 4, 8, 12, 16, and 20 cm of PE and 0, 3, and 6 cm of PVC, respectively. The contrast agent containers were made of PE, with an internal width of 0.85 cm and external width of 1 cm. They were filled with Visipaque (GE Healthcare) iodine contrast agent and water solution with concentrations of 0, 16, and 32 mg I/ml.

The energy thresholds of the detector were set at energies approximately equal to 15, 21, 31, 37, 43, 50, 59, and 68 keV, which produced an approximately equal number of counts in each bin for an air scan. Due to the energy response of silicon, this coincides with having narrow bins at lower energies which is beneficial for differentiating calcium from iodine. Calibration measurements were then acquired with all 54 different possible combinations of PE, PVC, and contrast agent placed between the x-ray source and the detector. A forward model for the response of each bin in each detector channel was found by fitting a parametric forward model of the following form [36] to the acquired calibration data:

$$ \lambda \left({A}_{\mathrm{PE}},{A}_{\mathrm{PVC}},{A}_{\mathrm{I}}\right)=\sum \limits_{k=1}^K{w}_k\exp \left(-{d}_k\sum \limits_{\alpha \in \left\{\mathrm{PE},\mathrm{PVC},\mathrm{I}\right\}}{f}_{\alpha}\left({E}_k\right){A}_{\alpha}\right), $$

where *w*_*k*_, *d*_*k*_, *E*_*k*_, and *K* are model parameters, *f*_*α*_(*E*) are tabulated linear attenuation coefficient data for each of the basis materials [38], and *A*_*α*_ denote the basis material pathlength. The model parameters were found by choosing *K* = 3 and minimizing the negative Poisson log-likelihood

$$ \underset{w_k,{d}_k,{E}_k}{\arg \kern0.15em \min}\sum \limits_{i=1}^N\lambda \left({\boldsymbol{A}}_i\right)-{y}_i\log \kern0.15em \lambda \left({\boldsymbol{A}}_i\right), $$

where ***A***_*i*_ = (*A*_PE, *i*_, *A*_PVC, *i*_, *A*_I, *i*_) denotes the pathlengths of each basis material in the *i*th calibration phantom data point and *y*_*i*_ denotes the corresponding mean number of counts measured by a specific detector channel and bin. The forward models were further improved by fitting a low-degree polynomial *C*(***A***) to the resulting set of relative errors *y*_*i*_/*λ*(***A***_*i*_), with the final forward model being given by $\overset{\sim }{\lambda}\left(\boldsymbol{A}\right)=\lambda \left(\boldsymbol{A}\right)C\left(\boldsymbol{A}\right)$.

### Material decomposition

Material decomposition of the projection data was performed by the maximum-likelihood method assuming Poisson statistics [39], for which the estimate of ***A*** for an observed set of bin counts *y*_1_, …, *y*_8_ is given by

$$ {\boldsymbol{A}}^{\ast }=\underset{\boldsymbol{A}}{\arg \kern0.15em \min}\sum \limits_{j=1}^8{\overset{\sim }{\lambda}}_j\left(\boldsymbol{A}\right)-{y}_j\log \kern0.15em {\overset{\sim }{\lambda}}_j\left(\boldsymbol{A}\right), $$

where ${\overset{\sim }{\lambda}}_j\left(\boldsymbol{A}\right)$ is the bin response function for bin *j*, determined from the calibration data as described above. Additional measurements for selected calibration data points were acquired for the purpose of validation.

### Preparation of hearts from deceased patients

Hearts from two individuals were obtained. An approximately 20-mm-thick slice containing calcified plaque was excised from each heart and placed in 4% formalin for 1 week. During that time, a custom-3D-printed (Ultimaker 2 Extended+, Ultimaker BV) phantom holder was made to enable imaging sessions in both vertical and horizontal beam geometry. The phantom holder was cylindrical with a diameter of 14 cm, with a 0.5-cm cutout on one side. After fixation, the heart samples were removed from the formalin, the coronary arteries were sutured, and a mix of isotonic sodium chloride and Visipaque (GE Healthcare) 270 mg I/ml (1:5) was prepared. That solution was then mixed with gelatin (10 g/100 ml) and injected into the coronary arteries. This gives a concentration of approximately 5 mg I/ml which is in the clinically relevant range. The choice of gelatin is suboptimal since it is water-like and not a perfect representation of blood in terms of CT value, but was selected in order to fixate the contrast agent solution and enable imaging in both vertical and horizontal beam geometry at multiple sites.

### Data acquisition and image formation

The hearts were scanned on a 1st-generation state-of-the-art dual-energy system, GE Discovery CT750 HD (GE Healthcare), hereafter referred to as the clinical scanner. We used an 80/140 switched kVp dual-energy chest scan protocol (GSI 15) in helical mode with a pitch of 1.375, an exposure time of 0.6 s, a tube current of 640 mA, a focal spot size of 1.2 mm, beam collimation of 40 mm, and a large bowtie filter. The hearts were then scanned in the bench-top setup using 360 degrees of rotation, 4000 views, and a total current-time product of 189 mAs. Data from three detector slices were added to obtain a slice thickness of 0.65 mm in the isocenter, approximately matching that of the clinical scanner.

The native dual-energy algorithms of the clinical scanner were used to reconstruct a 70-keV synthetic monoenergetic image, which was the standard setting for the protocol at the clinical site, as well as a water image in a water-iodine decomposition and a water image in a water-calcium decomposition. These images were chosen as the best representatives of a virtual non-contrast image and a virtual non-calcium image. The images were reconstructed using a standard kernel, which was the highest-resolution kernel available for spectral imaging on the system.

Material decomposition of the PCD data was performed using the described maximum-likelihood method, resulting in three-material sinograms. From these, material-specific images were reconstructed using filtered back-projection, using both a high-resolution Ram-Lak kernel and a smoother cosine kernel. The high-resolution images were used to create a synthetic monoenergetic image, and the smoother images were used to create a virtual non-contrast image and a virtual non-calcium image. All images were created using linear blending of the basis images. The weights for the synthetic monoenergetic image were chosen to minimize the variance in the image. The virtual non-contrast and virtual non-calcium images were created with the same weights but excluding either the iodine or calcium image. Ring artifacts were removed in the basis images in such a way that the synthetic monoenergetic image was unaffected.

### Dose comparison

Since the standard method for measuring dose using CTDI phantoms is not well suited for comparing systems with different scan geometries and beam collimations, we instead computed an estimate of the ratio of dose-length products (DLP) used to form the images in the two systems. Since DLP = (eff mAs · dose rate · slice thickness), the DLP ratio is given by

$$ \frac{\mathrm{DL}{\mathrm{P}}_{\mathrm{bench}-\mathrm{top}}}{\mathrm{DL}{\mathrm{P}}_{\mathrm{clinical}}}={r}_{\mathrm{dose}\ \mathrm{rate}}\cdotp {r}_{\mathrm{eff}\ \mathrm{mAs}}\cdotp {r}_{\mathrm{slice}\ \mathrm{thickness}}, $$

where *r*_dose rate_ is the ratio of dose rates (in mGy/mAs) in the systems, *r*_eff mAs_ is the ratio of effective current-time products used in the scans, and *r*_slice thickness_ is the ratio of reconstructed slice thicknesses. The peak dose rate in free air in the bench-top setup was measured using an ionization chamber (PTW microLion, PTW). Since free-in-air dose rate data is not available for the clinical scanner, we used measured data from a 120-kV protocol with 20-mm collimation and a large bowtie filter on a single-energy CT scanner, similar to the clinical scanner, as a substitute [40]. The measured rates were rescaled to isocenter and the resulting ratio yielded *r*_dose rate_.

## Results

### Material decomposition

**Fig. 2:**
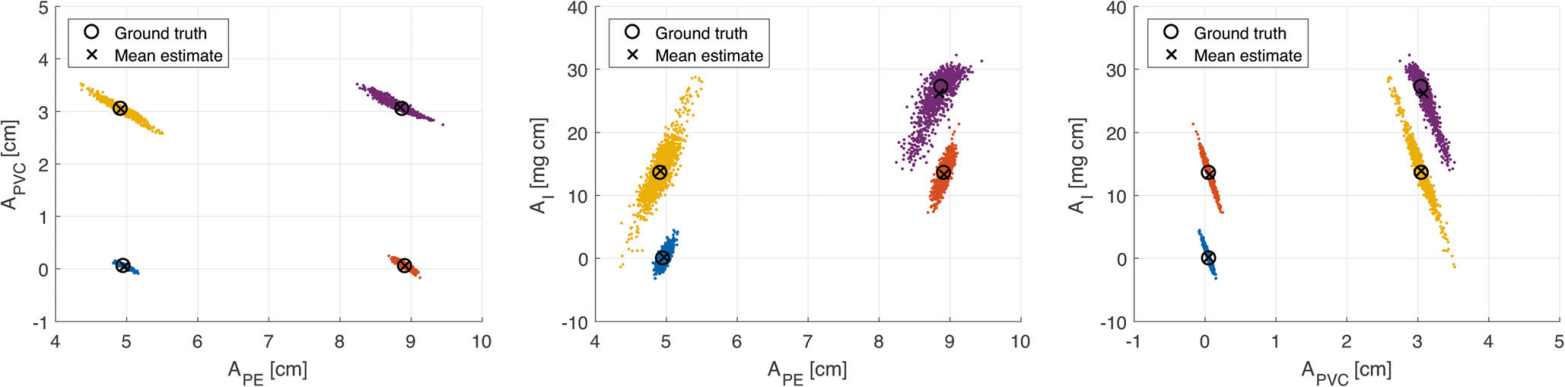
Results from applying the material decomposition algorithm to four selected calibration data points. Each dot is the result of the material decomposition algorithm on the photons acquired in one view/projection. The spread of each point cloud is due to the statistical uncertainty due to photon noise. Each plot depicts the same estimates viewed from a different axis. The labels *A*_PE_, *A*_PVC_, and *A*_I_ denote pathlengths through the phantom of the corresponding material. The mean over frames and true pathlength values are marked in the images. These results are also compiled in Table 1

The results from applying the material decomposition method to selected calibration data points are presented in Fig. 2, where the spread of each point cloud represents the statistical uncertainty due to photon noise. Quantification of systematic bias and noise for the selected calibration data points are presented in Tables 1 and 2. The results in Table 1 show that the systematic bias in the pathlength estimates is small, with a maximum absolute bias of 0.2 cm for PE, 0.1 cm for PVC, and 1 mg for 1 cm of iodine, and that an accurate estimate of iodine concentration can be achieved in a background of both PE and PVC.

**Table 1:** Material decomposition bias. Measured bias resulting from applying the material decomposition to selected points in the calibration data set. The ground truth value represents the actual pathlength of each material, taking into account the estimated fraction of water in the contrast agent solution expressed as a combination of PE and PVC. The presented estimate is the sample mean of estimates for each measurement and pixel in the detector

Sample point	*A*_PE_ [cm] Ground truth / estimate	*A*_PVC_ [cm] Ground truth / estimate	*A*_I_ [mg cm] Ground truth / estimate
1	5.0 / 5.0	0.1 / 0.1	0.0 / 0.0
2	8.9 / 8.9	0.1 / 0.1	13.6 / 13.3
3	4.9 / 4.9	3.1 / 3.0	13.6 / 13.9
4	8.9 / 8.8	3.1 / 3.1	27.2 / 26.2

**Table 2:** Material decomposition noise. Measured noise resulting from applying the material decomposition method on selected points in the calibration data set. The three left columns present the sample standard deviation of the noise for each material estimate, and the three right columns present the correlation coefficients between the different material estimates

Sample point	${\sigma}_{A_{\mathrm{PE}}}$ [cm]	${\sigma}_{A_{\mathrm{PVC}}}$ [cm]	${\sigma}_{A_{\mathrm{I}}}$ [mg cm]	${\rho}_{A_{\mathrm{PE}},{A}_{\mathrm{PVC}}}$	${\rho}_{A_{\mathrm{PE}},{A}_{\mathrm{I}}}$	${\rho}_{A_{\mathrm{PVC}},{A}_{\mathrm{I}}}$
1	0.05	0.04	1.13	− 0.90	0.75	− 0.96
2	0.07	0.06	2.06	− 0.91	0.80	− 0.98
3	0.16	0.13	3.92	− 0.96	0.87	− 0.97
4	0.17	0.12	3.30	− 0.95	0.79	− 0.93

### Heart images

**Fig. 3:**
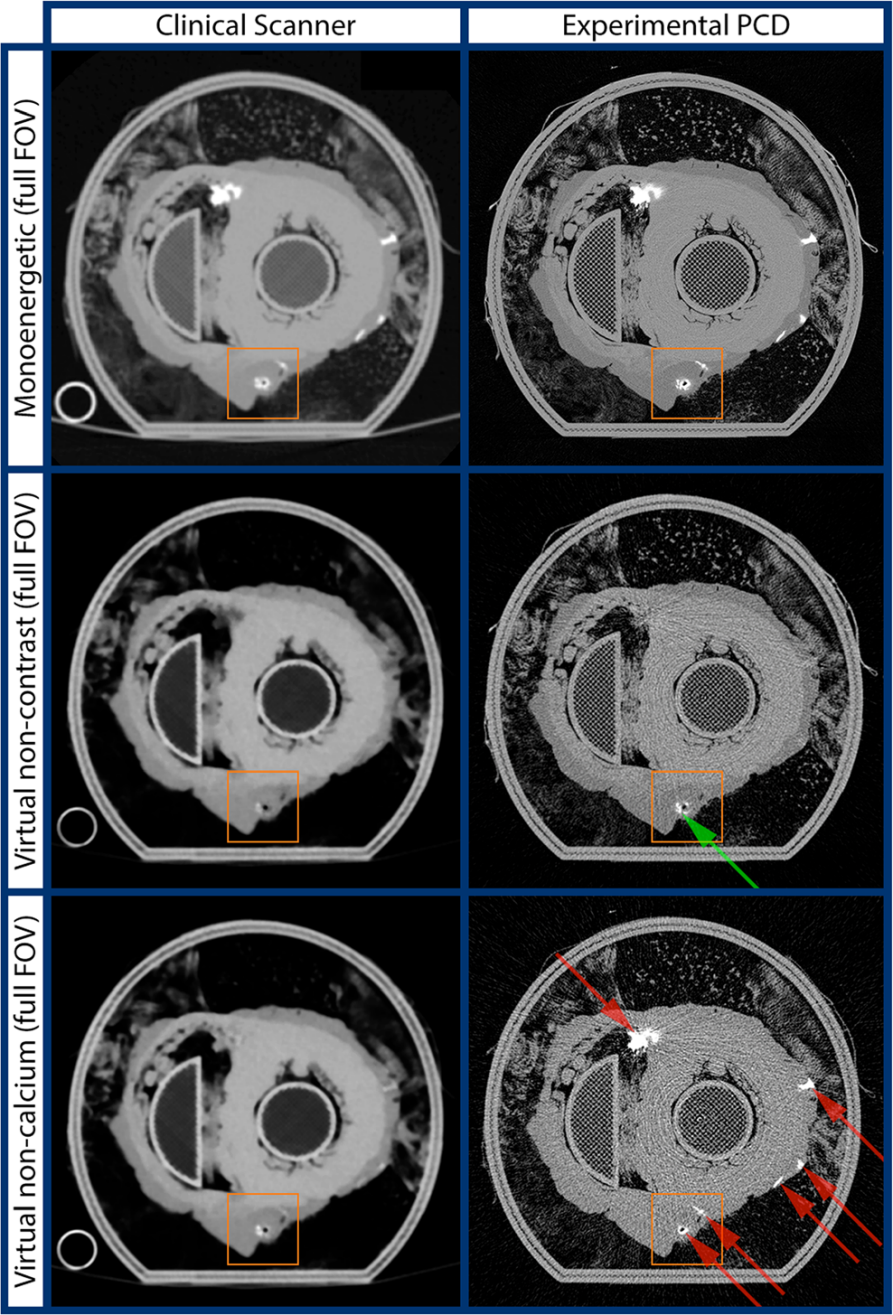
Images in full field of view. Row 1: synthetic monoenergetic images. Row 2: virtual non-contrast images. Row 3: virtual non-calcium images. Left column: the clinical scanner. Right column: experimental photon-counting detector (PCD). Red arrows indicate presence of iodine. Green arrows indicate presence of calcifications. Orange box indicates region of interest presented in Fig. 4

**Fig. 4:**
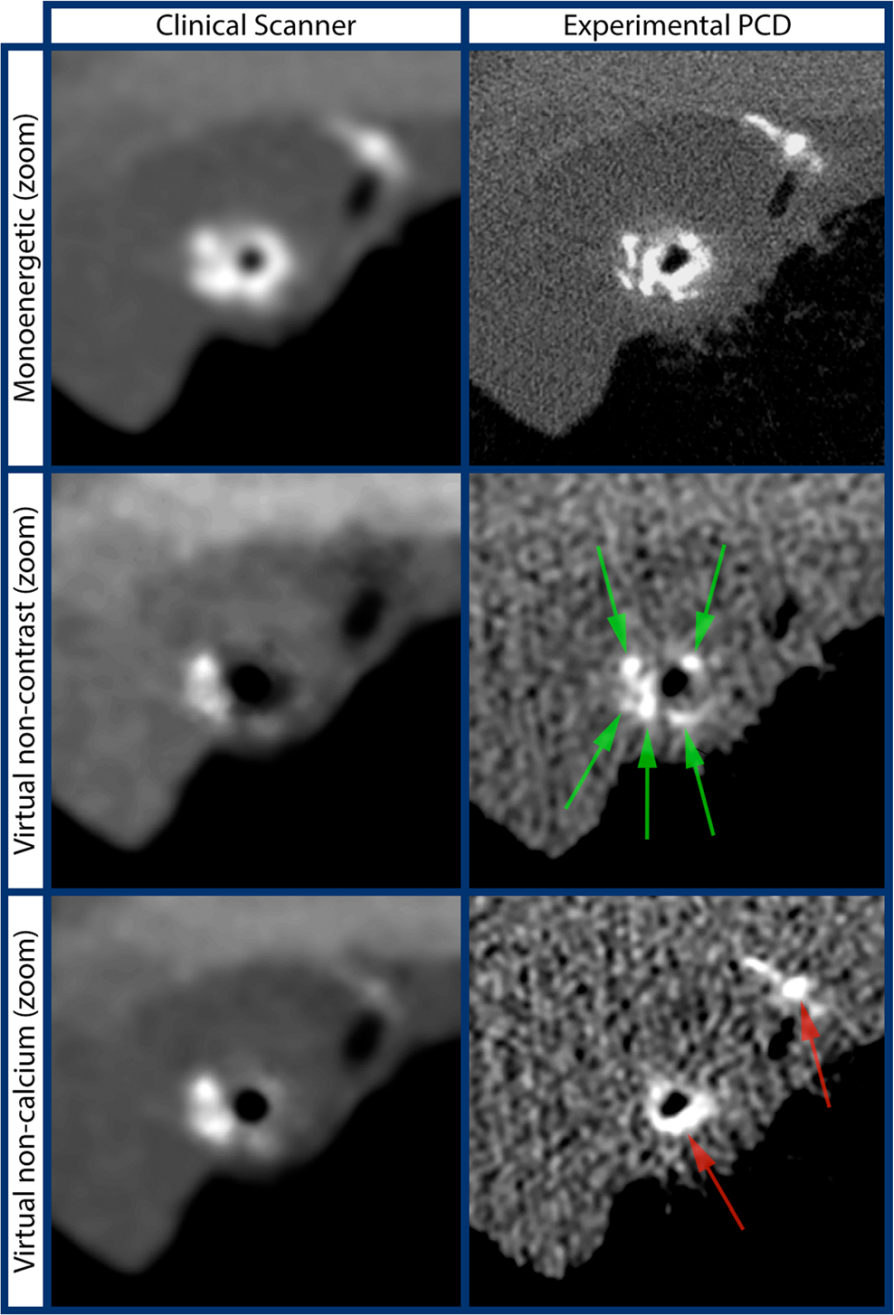
Zoom of region of interest (orange box) shown in Fig. 3. Row 1: synthetic monoenergetic images. Row 2: virtual non-contrast images. Row 3: virtual non-calcium images. Left column: the clinical scanner. Right column: experimental photon-counting detector (PCD). Red arrows indicate presence of iodine. Green arrows indicate presence of calcifications

Synthetic monoenergetic, virtual non-contrast and virtual non-calcium images of the heart sample in full field of view are shown in Fig. 3. Detailed images of a vessel containing both a calcified plaque and contrast agent are shown in Fig. 4. High concentrations of iodine and calcium are indicated with red and green arrows respectively. The locations indicating iodine in the image coincide with vessels known to contain iodine.

### Dose comparison

The DLP ratio was computed as follows. The peak dose rate in free air measured in the bench-top setup was 0.19 mGy/mAs in the isocenter. In a conventional single-energy CT scanner, similar to the clinical scanner, with a 120-kV protocol, it was measured to 0.19 mGy/mAs at the same distance [40]. The resulting ratio *r*_dose rate_ = 1.0 indicates that the beam quality in the bench-top setup was similar to that of the conventional CT scanner. The ratio of effective current-time products was *r*_eff mAs_ = 189/(384/1.375) = 0.68, and the ratio of reconstructed slice thicknesses was *r*_slice thickness_ = 0.65/0.625 = 1.04. This gives a DLP ratio of

$$ \frac{\mathrm{DL}{\mathrm{P}}_{\mathrm{bench}-\mathrm{top}}}{\mathrm{DL}{\mathrm{P}}_{\mathrm{clinical}}}=1.0\cdotp 0.68\cdotp 1.04=0.70 $$

Assuming that the dose rate of the clinical scanner is similar to that of a conventional single-energy scanner, the DLP used to form the PCD images was thus estimated to be approximately 70% of that used for the clinical scanner.

## Discussion

The PCD images have noticeably higher spatial resolution than the images from the clinical scanner. This is explained both by the smaller pixel size of the PCD (0.270 mm compared to 0.625 mm in the isocenter) as well as the smaller focal spot of the bench-top setup (0.4 mm compared to 1.2 mm). The contribution from each of them to the resolution improvement can be estimated by approximating the combined blur from the focal spot size and detector pixel size as $B\left(F,D\right)=F\left(1-\frac{1}{M}\right)+\frac{D}{M}$, where *F* is the focal spot size, *D* is the detector pixel size, and *M* is the magnification, inserting values yields that

$$ \frac{B\left({F}_{\mathrm{clinical}},{D}_{\mathrm{bench}-\mathrm{top}}\right)}{B\left({F}_{\mathrm{clinical}},{D}_{\mathrm{clinical}}\right)}=0.7,\kern0.5em \frac{B\left({F}_{\mathrm{bench}-\mathrm{top}},{D}_{\mathrm{bench}-\mathrm{top}}\right)}{B\left({F}_{\mathrm{clinical}},{D}_{\mathrm{clinical}}\right)}=0.39.\kern0.5em $$

The total reduction in blur is thus approximately 61%, of which 30% is due to the reduced pixel size and 31% is due to the reduced focal spot size. The PCD images are also noisier than the images from the clinical scanner, which is expected due to its higher spatial resolution and the reduced dose. It is well known that a detector with smaller pixels allows for reconstructing images with higher spatial resolution at the expense of increased noise, but it also allows for reconstructing images with the same resolution and lower noise than a detector with larger pixels [41]. The PCD images show little to no overlap between the iodine and calcium signals, most clearly seen in the virtual non-contrast and virtual non-calcium images in Fig. 3 and Fig. 4. In comparison, the spectral images from the clinical scanner show significant overlap. The is explained by the well-known fact that when DECT is used to perform two-material decomposition when there are more than two materials in the object, there will be bias in the basis material estimates [4]. Although quantitative accuracy is hard to assess in a clinical specimen due to the difficulty in obtaining ground truth, the fidelity of the proposed method is validated by the agreement between the virtual non-calcium and virtual non-contrast images with known positions of vessels containing iodine. The results presented in Table 1 and Fig. 2 further validate that estimates with low bias of all three materials can be achieved. The results in Table 2 show negative noise correlation between estimates of PE and PVC as well as PVC and iodine, whereas the noise correlation between estimates of PE and iodine is positive. This is explained by the fact that the linear attenuation of iodine is best approximated by a positive part of PVC and a negative part of PE. The computed dose estimates, although approximative, indicate that it is not a large increase in dose that allows the improved differentiation of calcium and iodine. While dose reduction is one of the promises held by PCD, we refrain at this point from making any claims regarding dose savings given that the bench-top setup might not accurately reflect clinical usage. Although ring artifact correction has been applied, there are some residual ring artifacts visible in the experimental PCD images, possibly due to changes in pixel response during the time of measurement or imperfect calibration. In this particular experimental setup, the exposure times were extremely long and experiments were performed in an environment without temperature control, both circumstances increasing the sensitivity for ring artifacts. In a clinical system, both temperature and the electrical environment are highly controlled, leading to better detector stability and therefore also reduced ring artifacts. Improvements in the calibration and ring artifact correction methods could also further suppress ring artifacts.

PCDs have been used in previous studies for excised atherosclerotic plaque imaging, using both maximum-likelihood-based approaches [32] and constrained image-based approaches [27, 28]. The constrained image-based approach in [28] has also been successfully used for dual-contrast cardiac and abdominal imaging of canine models [33, 34], with both iodine and calcium as basis materials. Unconstrained three-material decomposition has also been demonstrated in previous studies [21**,** 26]. In this work, we demonstrate for the first time the usefulness of unconstrained three-material decomposition for imaging of anatomical samples with a silicon detector developed for high flux imaging. It is a well-known fact that single-energy CT scanners with energy-integrating detectors over-estimate plaque volume by up to 400% [42] due to calcium blooming artifacts. DECT has the potential to improve upon this, but current DECT material decomposition methods do not allow the radiologist to accurately differentiate between iodine and calcium. This makes assessing the actual diameter of the stenosis impossible, and any error in the estimation of the stenosis grade in a calcified plaque will have implications for all secondary analysis of pressure gradients and risks. The performance of current DECT material decomposition methods is expected and a well-known phenomenon within the clinical routine work where it is applied. At the same time, there is substantial evidence for the use of coronary CT-angiography as a method to provide prognostic data for low-risk patients, albeit with lower positive predictive values for a myocardial event [10]. To further enhance the predictive values, new techniques need to be developed. A clinical system based on the proposed PCD has the potential for at least two practical improvements: higher spatial resolution stemming from smaller pixels and improved separation of calcium and iodine due to more available spectral information. This could make it possible to further differentiate and characterize different plaques in order to find new risk factors. Despite recent claims to the contrary [6], our results show that it is possible to differentiate iodine and calcium with limited overlap using projection-based material decomposition with the experimental PCD. There are two important aspects of this result. The first is that the imaged object was of modest size, approximately 14 cm in diameter, which allows a high enough fraction of x-rays which are close to the k-edge of iodine in energy to escape the object unattenuated, enabling iodine to be differentiated from calcium. The second is that the spectral response of the experimental PCD preserves this information. Compared to other proposed PCD systems [6, 14–18], the system we propose has a technical advantage that allows for handling high count rates without degrading the energy resolution and spectral performance of the detector. The PCD used in this study has been shown to have an approximately linear response for incident fluences up to 90 Mcps/mm^2^ and a count loss of 30% at a fluence of 485 Mcps/mm^2^ [23], with an estimated degradation of energy resolution of 0.6 keV and 3.4 keV at the respective fluences, based on measurements performed on a previous prototype [24].

A limitation of this study is that neither the focal spot size nor the tube current used in the bench-top setup is representative of clinical scans. A larger focal spot would reduce the achievable spatial resolution, and consequently, although the presented results are in line with the fact that improved spatial resolution is one of the promises of PCDs, further investigation is needed to determine how much of the presented resolution improvement can be retained in a clinical system. Also, differentiating iodine and calcium becomes more challenging with large patient anatomies [5]. Since the diameter of the imaged objects, 14 cm, is smaller than most patients, the results of this paper are not directly transferable to cardiac imaging in general without further study, but likely to other imaging areas where the anatomy is comparable in size to that of the heart phantoms, for instance neck- or pediatric imaging, where typical diameters are in ranges between 11 and 16 cm [43, 44]. Another limitation of this study was that only two heart samples were used. The data from the second heart sample showed similar results as the first but showed signs that the contrast agent had leaked between image acquisitions on the clinical scanner and in the bench-top setup, making a comparison difficult. Another limitation was that the generated basis images did not correspond exactly to water and calcium, since PE and PVC were used as basis materials. These materials were selected because they allow representing any material in the human body with positive coefficients. In the future, the calibration could be supplemented with additional measurements of water and hydroxyapatite, which would allow generating images more accurately representing water and calcium by performing a basis change from PE and PVC to water and calcium.

In conclusion, in this study, we have demonstrated that an experimental photon-counting silicon detector allows performing unconstrained three-material decomposition, thereby giving better separation of iodine and calcium than DECT. Further investigation is needed to determine how much of this improvement can be retained in a clinical system, but considering its previously shown count rate capability, these results show promise for future integration of this detector in a clinical CT scanner.

## Abbreviations

ASIC: Application-specific integrated circuit
DECT: Dual-energy computed tomography
DLP: Dose-length product
PCD: Photon-counting detector
PE: Polyethylene
PVC: Polyvinylchloride
